# Modulations in the Peripheral Immune System of Glioblastoma Patient Is Connected to Therapy and Tumor Progression—A Case Report from the IMMO-GLIO-01 Trial

**DOI:** 10.3389/fneur.2017.00296

**Published:** 2017-06-23

**Authors:** Paul F. Rühle, Nicole Goerig, Roland Wunderlich, Rainer Fietkau, Udo S. Gaipl, Annedore Strnad, Benjamin Frey

**Affiliations:** ^1^Department of Radiation Oncology, Universitätsklinikum Erlangen, Friedrich-Alexander-Universität Erlangen-Nürnberg, Erlangen, Germany; ^2^Research Unit of Radiation Cytogenetics, Helmholtz Center Munich, Neuherberg, Germany

**Keywords:** glioblastoma multiforme, radiochemotherapy, immunophenotyping, immune status, liquid biopsy, personalized medicine

## Abstract

Immune responses are important for efficient tumor elimination, also in immune privileged organs such as the brain. Fostering antitumor immunity has therefore become an important challenge in cancer therapy. This cannot only be achieved by immunotherapies as already standard treatments such as radiotherapy and chemotherapy modify the immune system. Consequently, the understanding of how the tumor, the tumor microenvironment, and immune system are modulated by cancer therapy is required for prognosis, prediction, and therapy adaption. The prospective, explorative, and observational IMMO-GLIO-01 trial was initiated to examine the detailed immune status and its modulation of about 50 patients suffering from primary glioblastoma multiforme (GBM) or anaplastic astrocytoma during standard therapy. Prior to the study, a flow cytometry-based assay was established allowing the analysis of 34 immune cell subsets and their activation state. Here, we present the case of the first and longest accompanied patient, a 53-year-old woman suffering from GBM in the front left lobe. In context of tumor progression and therapy, we describe the modulation of the peripheral immune status over 17 months. Distinct immune modulations that were connected to therapy response or tumor progression were identified. *Inter alia*, a shift of CD4:CD8 ratio was observed that correlated with tumor progression. Twice we observed a unique composition of peripheral immune cells that correlated with tumor progression. Thus, following up these immune modulations in a closely-meshed manner is of high prognostic and predictive relevance for supporting personalized therapy and increasing therapy success.

**Clinical Trial registration**: ClinicalTrials.gov, identifier NCT02022384 (registered retrospectively on 13th of December, 2013).

## Introduction

Glioblastoma multiforme (GBM) is the most common primary malignant brain tumor which is very aggressive and almost always recurrent (1, 2). Despite the improvements in the multimodal therapy including surgery, radiotherapy (RT), and chemotherapy (CT), GBM is still associated with a poor median survival of only 13–16 months (1, 3) and a 5-year survival rate below 5% (1, 4, 5). Therefore, further therapy options, such as immunotherapy (6–8), are desperately needed. Even though access to the brain is highly regulated by the blood–brain barrier, immunotherapies might be very beneficial in GBM treatment as today it is commonly accepted that leukocytes migrate into the brain (9). Furthermore, it has been demonstrated that microenvironment of GBM drives chemo- and immunotherapy resistance (10) and that the dynamics of interactions of immune cells that pass the blood–brain barrier with glioblastoma cells contribute to glioblastoma pathogenesis and might serve as basis for therapy improvements (11).

Highly important for the successful application of immunotherapy together with radiochemotherapy (RCT) is the right time point for its inclusion and a previous estimation of individual therapy responses. Therefore, we initiated the IMMO-GLIO-01 study. The goal of this prospective, explorative, and observational study has been a detailed longitudinal evaluation of the immune status from patients suffering from GBM grade IV or anaplastic astrocytoma grade III in course of a standard RCT. Eventually, immune biomarkers should be defined that are connected to individual therapy response for translation into clinic. For this, liquid biopsies are mandatory since solid biopsies can only be taken at limited time points and, therefore, just reflect a snapshot of the disease (12). We, therefore, previously established a flow cytometry-based assay for detailed immunophenotyping of blood allowing the identification of 37 different cell subsets circulating the periphery and the characterization of their activation status without the need of preceding cell isolation (13).

Here, we present the immunological data of the first and longest accompanied patient in the IMMO-GLIO-01 study. We show that intensive RCT does not lead to general immune suppression, but rather to distinct immune modulations and that the latter might be linked to therapy and clinical response of the tumor.

## Methods

**Figure 1:**
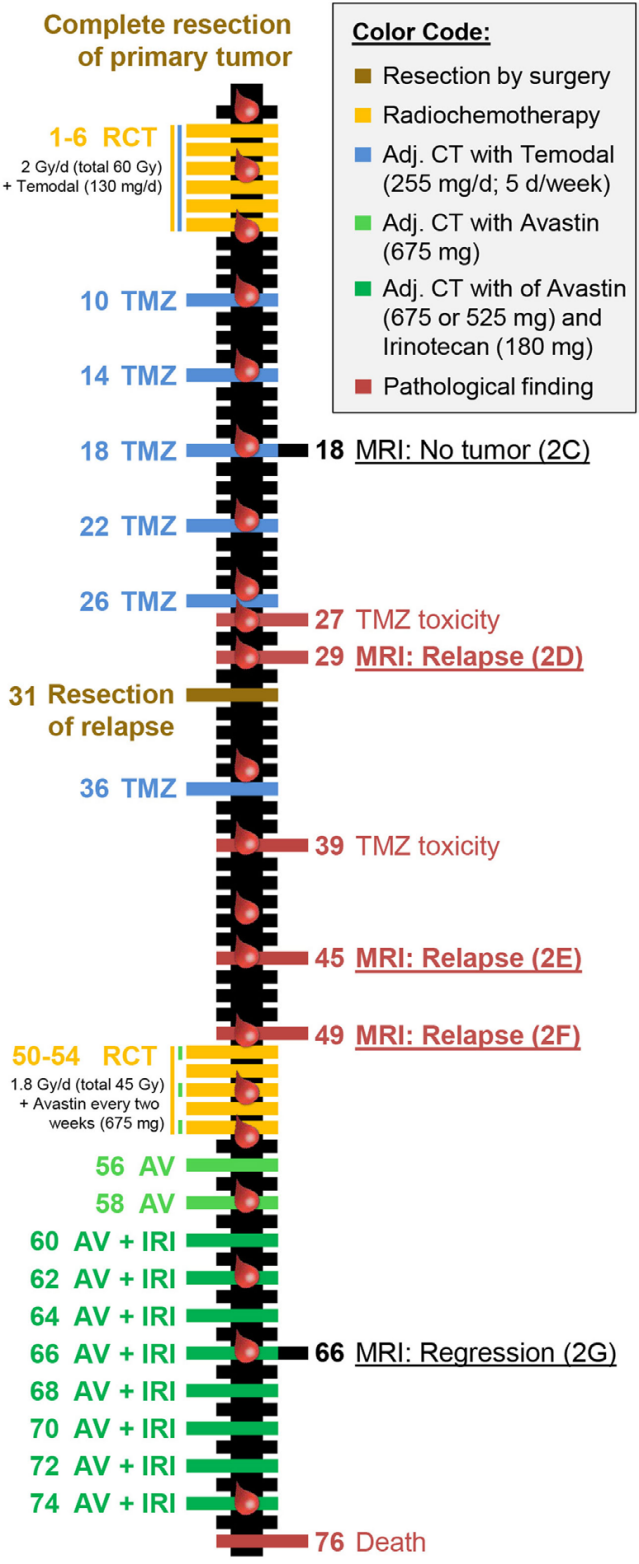
Individual therapy schedule and clinical response of the reported patient with glioblastoma. The time line depicts treatment (left side) and clinical response (right side) including pathological abnormalities and tumor progression. All numbers stated are in weeks with zero simultaneously being the starting point of radiochemotherapy and the evaluation of the immune status (indicated by red blood drops). The colors represent treatments or therapy outcomes according to the color code presented in the top right corner. This color code was also used for data presentation in Figure 3. The magnetic resonance imaging scans directly refer to the according image in Figures 2C–G.

The IMMO-GLIO-01 study (clinical trials ID: NCT02022384) is a prospective, explorative, and observational trial at the Department of Radiation Oncology at the Universitätsklinikum Erlangen and has been performed in accordance with the Declaration of Helsinki. Ethical approval was granted from the ethics committee of the University Erlangen-Nürnberg Medical School. The patient signed a declaration of consent to participate in the study and provided a written consent that the obtained data can be published as case report or as any other publication in a pseudonymised manner. Blood was drawn in a closely-meshed manner during course of tumor therapy as indicated in Figure 1 (red blood droplets). The blood was always taken before administration of therapeutics on that day avoiding misinterpretation by immediate short-time reactions. For all 21 blood samples, we performed detailed immunophenotyping (13). The staining and analysis of all samples were performed blinded.

## Presentation of the Case: Clinical Part

**Figure 2:**
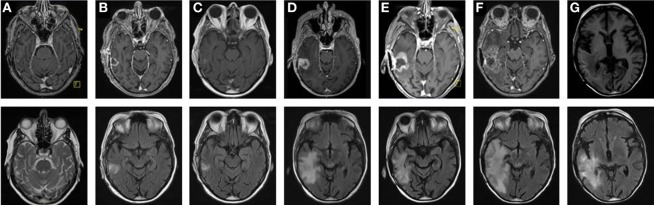
Tumor progression identified by consecutive magnetic resonance imaging (MRI) scans reveals transient therapy success and two relapses. MRI scans showing T1- (upper panel) and T2-weighted images (lower panel) of a glioblastoma multiforme in the right temporal lobe, from primary diagnosis through the different stages of tumor progression. After primary diagnosis **(A)** and subsequent complete resection **(B)**, standard concomitant radiochemotherapy (RCT) followed by adjuvant chemotherapy with Temodal (TMZ) according to Stupp et al. was performed. The first follow-up MRI (3 months after RCT) showed no signs of tumor recurrence **(C)**. Two months later, an MRI was performed due to TMZ cytotoxicity. There were hints of tumor relapse **(D)**, which was histologically confirmed after the lesion had been completely removed by surgery. Yet only 2 **(E)** and 3 months **(F)** after this second resection, tumor progression was first suspected and then confirmed. Thus, a conversion of therapy was decided, the therapy of choice being secondary radiotherapy combined with Avastin. This led to tumor regression as observed during the 3-month follow-up during aftercare with Avastin and irinotecan **(G)**.

The first patient included in the IMMO-GLIO-01 study was a 53-year-old woman suffering from right temporal GBM grade IV (Figure 2A). She was recruited into the study directly after complete resection of the primary tumor (Figure 2B) and then received standard RCT according to Stupp et al. (3) (6 weeks, 60 Gy in 2 Gy fractions, daily 130 mg Temodal (TMZ); therapy scheme presented in Figure 1). Then, therapy was continued as adjuvant CT with TMZ (255 mg/day) until the fifth course (week 26) without any neurological or pathological complications [magnetic resonance imaging (MRI) presented in Figure 2C]. But in week 27 the patient complained of loss of appetite and nausea. Further examinations revealed an increase of transaminases to grade III–IV and a hepatic steatosis. Consequently, MRI was performed (week 29) depicting signs of a relapse (Figure 2D), which was later confirmed by histological examination. The interdisciplinary tumor board decided to conduct a second surgery (in week 31) and the relapsed tumor was successfully removed. As afterward the patient did not show any further clinical complications, the TMZ therapy was pursued with reduced dose (80%, week 36). Despite dose reduction of TMZ, the transaminase levels again increased to grade III (week 39). Consequently, TMZ therapy was stopped.

The next routine MRI was conducted in week 45 (3 months after surgery) and week 49 and revealed a new relapse (Figures 2E,F), but again this was paired without any neurological findings. This time, the interdisciplinary tumor board decided for a conversion of therapy to Avastin (bevacizumab) targeting vascular endothelial growth factor (VEGF), which had been approved for the treatment of recurrent gliomas. The RCT in combination with Avastin (5 weeks: 45 Gy in 1.8 Gy fractions, biweekly 675 mg Avastin) was followed by an adjuvant CT with biweekly applied Avastin (week 56 and week 58) or Avastin in combination with Irinotecan (from week 60 on). The success of this therapy conversion became visible in the next routine MRI (week 66) that showed a regression of the tumor relapse (Figure 2G). During Avastin therapy, the patient never showed any neurological or pathological complications, however, the patient died in week 76 in succession of an epileptic seizure as complication of the disease. Another fact worthy of note is that the patient did not have any infections during complete therapy and did not take any steroids, except directly after surgery.

## Presentation of the Case: Immune Status

**Figure 3:**
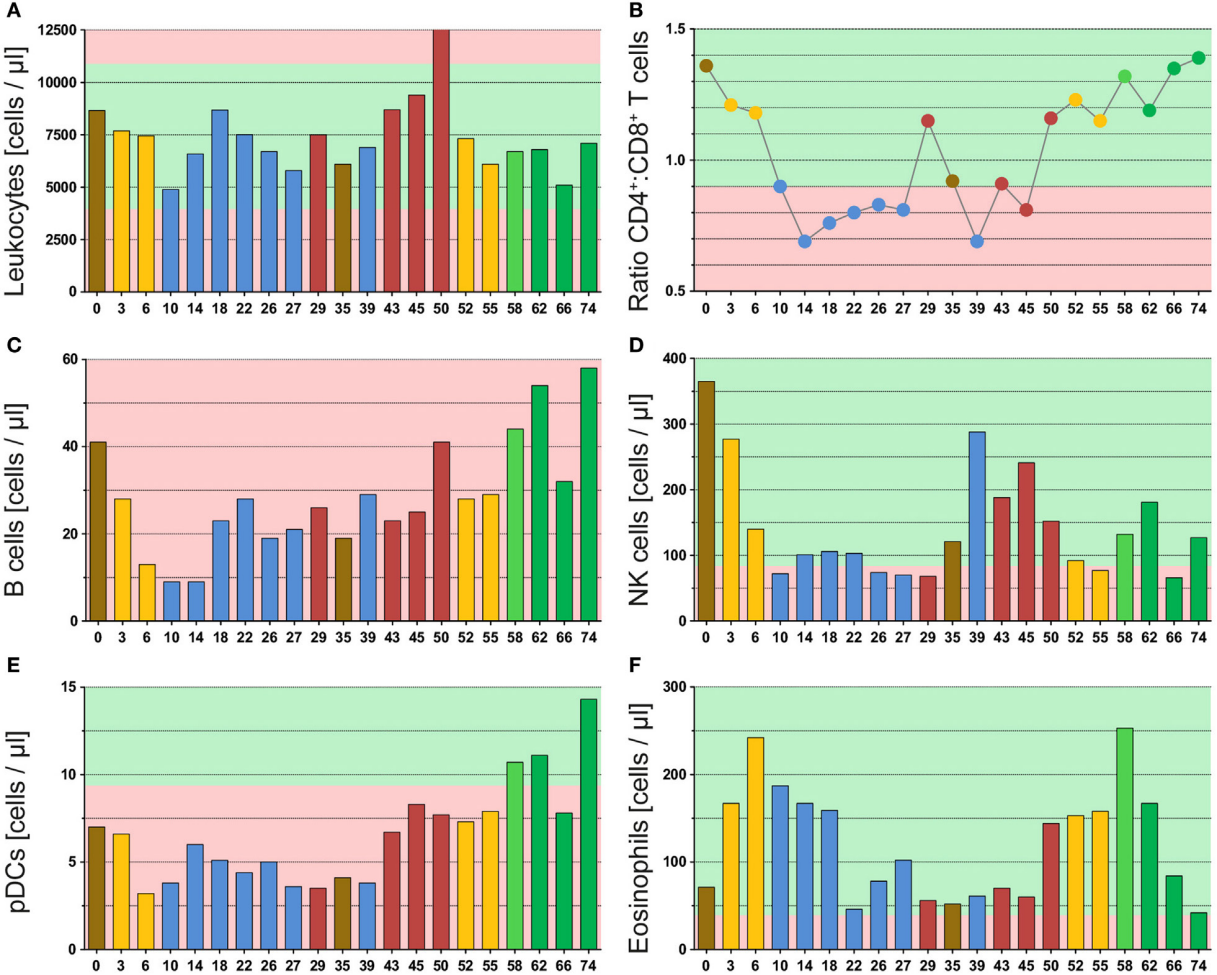
Multimodal therapy of glioblastoma has high impact on immune cells of the peripheral blood. The graphs of **(A)** and **(C–F)** display the absolute cell counts of immune cells or immune cell subsets in the peripheral blood, and in **(B)**, the ratio from CD4^+^ helper T cells to CD8^+^ cytotoxic T cells is displayed. All immune cells were determined by a multicolor flow cytometry-based assay and directly detected in whole blood samples. The blood was always drawn prior to administering the drug or radiotherapy on the respective day. The color code represents the last applied therapy or pathological finding (red) and was applied according to Figure 1. The green background marks normal values and the red one deviation from it.

The leukocyte count varied during the whole therapy, but never strikingly dropped below the normal range (Figure 3A). This emphasizes that the immune system is not destroyed during intensive anticancer RCT of glioblastoma. Yet, the leukocyte count decreased following RT in both applications (yellow bars), but recovered afterward. Besides, we observed a strong increase in the leukocyte count after discovery of the second relapse (red bars, week 45 and week 50), which especially was caused by neutrophils (Figure S1A in Supplementary Material).

The investigation of T cells revealed a strong temporary decrease following RT from 1,664 to 753 cells/μl (second RT: 1,488 to 737 cells/μl), but they recovered fast and then varied without distinct patterns during the remaining therapy (Figure S1B in Supplementary Material). In contrast, we found a connection between therapy and T cell subsets, which is presented as ratio of CD4^+^ T helper versus CD8^+^ cytotoxic T cells (Figure 3B). During administration of TMZ (blue dots), a strong shift toward cytotoxic T cells was observed. This was reversed in both relapse situations (red dots) and not observed following Avastin therapy (green dots). In this context, RT (yellow dots) might be inhibitory on or delaying the TMZ effect as during initial RCT (combined with TMZ) a small decrease in the CD4:CD8 ratio was identified. During the second RCT (combined with Avastin) no influence of RT on the CD4:CD8 ratio was seen.

The Avastin therapy improved the B cell count (Figure 3C: green bars), which was very low during the complete observation period (<40 cells/μl) and did not rise before Avastin administration. As observed for T cells, the B cell count dropped following initial RCT (from 41 to 13 cells/μl), but recovered later on, even this required more time. In contrast to T cells, the second RCT in combination with Avastin had only marginal impact on the B cell count suggesting a protective or beneficial role for Avastin on B cells.

The initial RT, likewise, had a very strong effect on NK cells which dropped from 365 to 140 cells/μl and remained fairly low during the complete period of adjuvant TMZ administration (Figure 3D: week 0–29). Following TMZ discontinuation and surgery, the NK cells increased again. Yet, the second RT induced a strong decline from 152 to 77 cells/μl. But in contrast to adjuvant TMZ administration, NK cells could recover during Avastin therapy (green bars) even thus they did not reach the initial level.

Also striking was the pattern of plasmacytoid dendritic cells (pDC). These immune cells also decreased during initial RCT (Figure 3E: from 7 to 3 cells/μl) and remained low during adjuvant TMZ administration, but increased heavily during Avastin intake (11–14 cells/μl). The pattern was very similar to that of B cells.

Furthermore, we found a surprising enhancement of eosinophils by RT, which was observed following both applications (Figure 3F: yellow bars). However, the second increase was already detected before start of RT, namely after detection of the second relapse in week 50. During that time point likewise neutrophils (Figure S1A in Supplementary Material) and B cells (Figure 3C) were conspicuously elevated.

Besides, the 29 week-time point (detection of the first relapse) was noticeable as an elevation of monocytes (Figure S1C in Supplementary Material) and neutrophils (Figure S1A in Supplementary Material) was observed. Moreover, the CD4:CD8 ratio was extremely reversed in direction of CD4^+^ T helper cells in both relapse situations (Figure 3B). This suggests a connection of these immune parameters to clinical response as they correlate with tumor progression monitored by MRI.

## Discussion

The IMMO-GLIO-01 study was initiated to estimate whether and in particular how multimodal therapy of brain tumors (Figure 1) including RCT impacts on the systemic immune status of the patients. Immunological patterns might be discovered that do relate to therapy application, response, and/or disease status. These patterns should be useful for estimation of individual responses (Figure 2), to stratify patients, and to estimate suited time points for inclusion of further immunotherapy (14). An intensive cytotoxic therapy as applied in the treatment of high-grade gliomas should certainly cause leukopenia (15). This was also observed in the presented case, especially following RT (Figure 3A). But, all immune subsets recovered in course of therapy and some even increased. This suggests the immune system to remain functionally active, as previously described for other tumor entities (16). Moreover, the immune system seemed to contribute to tumor elimination as we found certain immune modulations to be connected to tumor progression or therapy, even though, none of the administered therapeutics was particularly targeted against any immune cell.

The most striking pattern observed was the CD4^+^:CD8^+^ ratio that strongly shifted into direction of CD8^+^ cytotoxic T cells following TMZ administration. In the treatment of gliomas, TMZ is prescribed as it easily crosses the blood–brain barrier (17) and induces a G2-M arrest, especially in p53-mutated cells, which is true for most glioma cells. This leads to synchronization of the cell cycle in a radiosensitive phase (18). Our data suggest that TMZ additionally has an immunogenic potential supporting the stimulation of tumor killing CD8^+^ T cells in the periphery. However, this has to be proven by analyses of more patients within the IMMO-GLIO-01 trial. In fact, we measured a reversed setting during both relapse situations after the TMZ intake was stopped (Figure 3B). Yet, it remains unclear whether the decline of cytotoxic T cells led to a recurrence of the tumor or the progressing tumor induced a suppression of cytotoxic T cells. However, this ratio does not implicitly reflect the absolute count as the T cell count varied and in general was lower than before therapy. Anyhow, this distinct pattern might be beneficial for estimation of individual therapy responses and for identification of tumor progression. Besides, *in vitro* studies have shown that TMZ (as well as Avastin) has no negative effects on the functionality of CD8^+^ cytotoxic T cells (19) indicating not only a TMZ-related change in T cell numbers but also an enhanced antitumor immunity. In contrast, Grossman et al. (20) demonstrated that patients with a very low CD4^+^ T cell count induced by RT/TMZ have a lowered overall survival. These early deaths were not related to infections, but to an earlier tumor progression. Therefore, a drop of CD4:CD8 ratio might be adverse when it is due to very low CD4^+^ T cell count (<200 cells/μl) and not due to an increasing number of CD8^+^ cytotoxic T cells. Interestingly, during the period of dropping CD4:CD8 ratio, the innate NK cells were repressed (Figure 3D). These innate immune cells also play a major role in killing of tumor cells, but are less specific. We conclude that therapy-modified adaptive and innate immune responses are interconnected.

Besides leukopenia, RT caused an enhancement of eosinophils in both applications (Figure 3F). Eosinophilia in cancer treatment remains elusive and there are controversial reports associating it with good or bad prognosis (21–23). But these reports mainly investigated the level of eosinophils without a simultaneous evaluation of other infiltrative immune cells. In contrast, Carretero et al. (24) showed that eosinophils might indeed support tumor rejection, but only in the presence of tumor-specific CD8^+^ T cells. Moreover, activated eosinophils were not destructive cells releasing their cytotoxic granules, but modulated the tumor microenvironment by chemokine production and thus improved T cell infiltration. In addition, it was demonstrated that activated eosinophils preferentially migrated into tumor tissues. This attraction was probably achieved by release of damage-associated molecular patterns from the necrotic tumor cells (21). We now also detected an increase in eosinophils in the periphery, which only can be detected when whole blood samples are analyzed and, therefore, might be overseen in several routinely performed assays using isolated peripheral blood mononuclear cells as biomaterial. As RT is capable of enhancing tumor necrosis (25), this could explain the peripheral rise of eosinophils observed in this case report. Taken together, an elevated number of eosinophils followed by an increase in CD8^+^ T cells might be an immune marker for a beneficial therapy response.

The role of B cells in antitumor immunity remains debatable and, like eosinophils, so far was correlated with both improved (26, 27) and poor prognosis (28), which depends on balance of the various B cell subsets within the tumor microenvironment (29). On the one hand, these may enhance antitumor responses supporting CD8^+^ T cells by cytokine secretion or antigen presentation (30). But on the other hand, infiltrative B cells might even inhibit an effective induction of cytotoxic T cell response (31, 32). Thus, in the final analysis covering all patients, a careful comparison of the B cell subsets and individual therapy response will have to be performed. In the presented case, we found the B cells in a very low frequency, as described for many malignancies, and they did not recover until Avastin administration (Figures 1 and 3C). Avastin is an antibody designed to bind and neutralize VEGF activity. VEGF is overexpressed by most gliomas and plays an important role in angiogenesis, and initially its neutralization was believed to inhibit tumor vascularization and consequently interrupt its blood supply (33, 34). But more important, Avastin affects the malformed tumor vessels resulting in a normalization of them (33–35). This leads to not only reduced leakage and, therefore, lowered edema and intracranial pressure but also enhanced immune infiltration (36) as well as an influx of cytotoxic agents (35), such as irinotecan. Besides, the blocking of VEGF might also have a positive effect on dendritic cell (DC) number and maturation (37).

Irinotecan inhibits topoisomerase I and, therefore, prevents DNA from unwinding and consequently inhibits transcription and replication (38). Among others, myelosuppression and neutropenia are characterized side effects of irinotecan (39), but were not observed in the peripheral blood of the presented case (Figure 3). Although, here it should be stressed that direct short-term effects were not investigated as blood withdrawals were always conducted before drug administration pursuing the detection of general immune modulations. Anyhow, irinotecan might be involved in the conspicuous decline of many subsets in week 66 (Figure 3). Besides, Avastin administration might support the elevation of B cells and, therefore, probably pushes the humoral immunity. Concurrently, the pDCs increased (Figure 3E), which has often been associated with tumor progression and poor survival (40–42). However, this might only apply to inactivated pDC (43–45) as in contrast their activation by Toll-like receptor agonists provided a significant tumor rejection or even complete regression (46–48).

Finally, we aimed to find immune cell compositions that are useful for recognition of individual therapy responses and for estimation of suited time points for inclusion of further therapies. We found two blood withdrawal time points that were very striking and both of them were related to tumor progression. The first one was in week 29 directly after detection of the first relapse. Here, the CD4:CD8 ratio was extremely reversed and we found an elevation of neutrophils, monocytes, and myeloid DCs (mDCs) (Figures S1A,C,D in Supplementary Material). The second one was in week 50 directly before start of the second RCT after detection of the second relapse. Here, we observed a huge increase in neutrophils, as well as an elevation of B cells, eosinophils, and mDCs, and likewise the ratio of CD4:CD8 was reversed (Figure 3; Figure S1 in Supplementary Material).

## Concluding Remarks

We identified several connections between administration or response to radiochemo(immune)-therapy in GBM and variations in the immune cell compositions in the peripheral blood of the patient presented here. To pin distinct immune modulations to definite clinical responses, the obtained data including those related to the numerous additional immune cell subsets/parameters that have been determined by our immunophenotyping (13) will be analyzed in future in comparison to that of other patients of the IMMO-GLIO-01 study. We, however, already observed in the presented case modulations of activation markers in dependence on the therapy, such as CD38^+^ expression on T cells (Figure S2 in Supplementary Material) and C69 expression on B cells (Figure S3 in Supplementary Material). Merging these data will allow us to link immune matrices to clinical responses. Anyhow and importantly, here we identified distinct immune modulations in the peripheral blood on single patient level connected to tumor progression or therapy. This knowledge obtained by detailed and closely-meshed immunomonitoring without exclusion of distinct immune cell subsets by sample preparation might be meaningful for personalized therapy approaches of the future. However, this has to be proven by analyses of more patients within the IMMO-GLIO-01 trial. It might be applied to predict therapy responses or to support the identification of tumor progression. Immune monitoring should focus on liquid biopsies since closely-meshed monitoring is mandatory (see weekly alterations displayed in Figure 3) and liquid biopsies are easy to obtain, even in clinical routine (49). Booster immunization based on DC therapies (50) should be considered when beneficial CD8:CD4 ratio is detected. Since eosinophils orchestrate cancer rejection by normalizing tumor vessels and enhancing infiltration of CD8^+^ T cells (24) at these time points (in the presented case in weeks 10–18), therapeutically activation of eosinophils might emerge as a promising tool for the improvement of clinical cancer immunotherapy.

## Ethics Statement

Ethical approval (reference number: 266_13B) was granted from the ethics committee of the Medical School of the Friedrich-Alexander-Universität Erlangen-Nürnberg and the study has been performed in accordance with the Declaration of Helsinki. Every patient filled out written informed consent prior to inclusion into the study including consent that the obtained data can be published as case report or as any other publication in a pseudonymised manner.

## Author Contributions

PR performed most of the immunophenotyping and most of the data analyses and drafted the manuscript together with UG, BF, and AS. NG performed most of the clinical analyses (imaging data) together with AS and contributed to the writing of the manuscript. RW helped to perform the immunophenotyping and contributed to the data analyses. RF contributed to the design of the work. UG drafted and designed the study together with AS and BF, drafted the manuscript, and wrote it together with PR, AS, and BF. AS drafted and designed the study together with USG and BF, contributed to drafting of the manuscript, and performed the clinical analyses together with NG. BF drafted and designed the study together with UG and AS, drafted the manuscript, contributed to data analyses, and wrote the manuscript together with PR, AS, and UG.

## Conflict of Interest Statement

The authors declare that the research was conducted in the absence of any commercial or financial relationships that could be construed as a potential conflict of interest.

## Abbreviations

GBM, glioblastoma multiforme; CT, chemotherapy; RT, radiotherapy; RCT, radiochemotherapy; TMZ, temozolomide (brand name: Temodal); MRI, magnetic resonance imaging; VEGF, vascular endothelial growth factor; DC, dendritic cells; pDC, plasmacytoid DC; mDC, myeloid DC.
