# Supplementary material for: Modulations in the Peripheral Immune System of Glioblastoma Patient Is Connected to Therapy and Tumor Progression—A Case Report from the IMMO-GLIO-01 Trial
Source: Front Neurol. 2017 Jun 23;8:296. doi: 10.3389/fneur.2017.00296 (PMC5481307; doi:10.3389/fneur.2017.00296)
Supplement: Supplementary file 1 [file data_sheet_1.docx]

Supplementary Material

Modulations in the Peripheral Immune System of Glioblastoma Patient is Connected to Therapy and Tumor Progression – A Case Report from the IMMO-GLIO-01 Trial

Paul F. Rühle^1^, Nicole Goerig^1^, Roland Wunderlich^1,2^, Rainer Fietkau^1^, Udo S. Gaipl^1,#,*^, Annedore Strnad^1,#^ and Benjamin Frey^1,#^

*** Correspondence:** Prof. Dr. Udo S. Gaipl: [udo.gaipl@uk-erlangen.de](mailto:udo.gaipl@uk-erlangen.de)

**
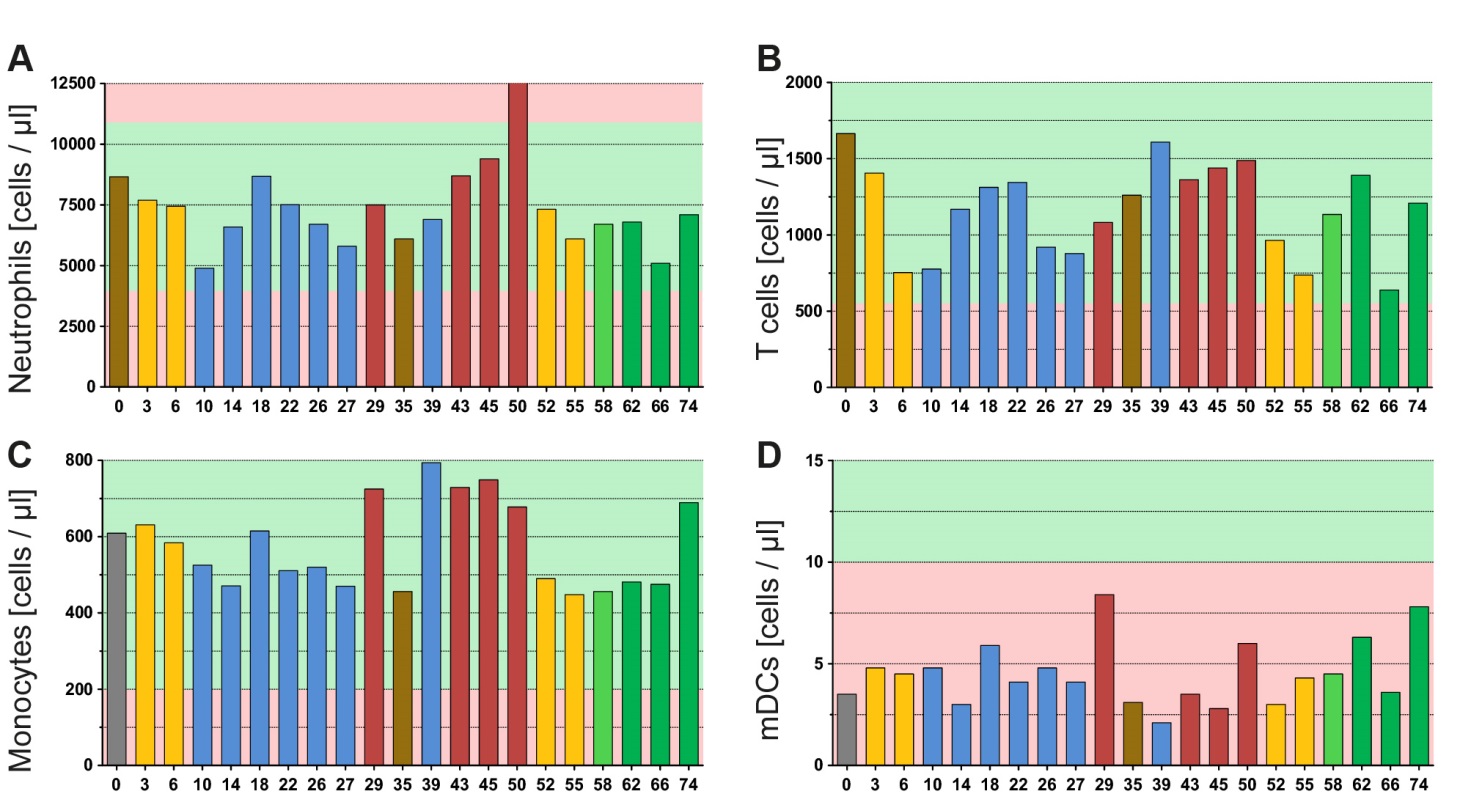
**

**Supplementary Figure 1: Multimodal therapy of glioblastoma has impact on several peripheral immune cells.** The graphs of **A-D** show the absolute cell counts of further immune cell subsets in the peripheral blood that were not presented in **Figure 3**. All immune cells were determined by a multicolor flow cytometry-based assay and directly detected in whole blood samples. The blood was always drawn prior to administering the drug or radiotherapy on the respective day. The color code represents the last applied therapy or pathological finding (red) and was applied according to **Figure 1**. The green background marks norm values and the red one deviation from it.

**
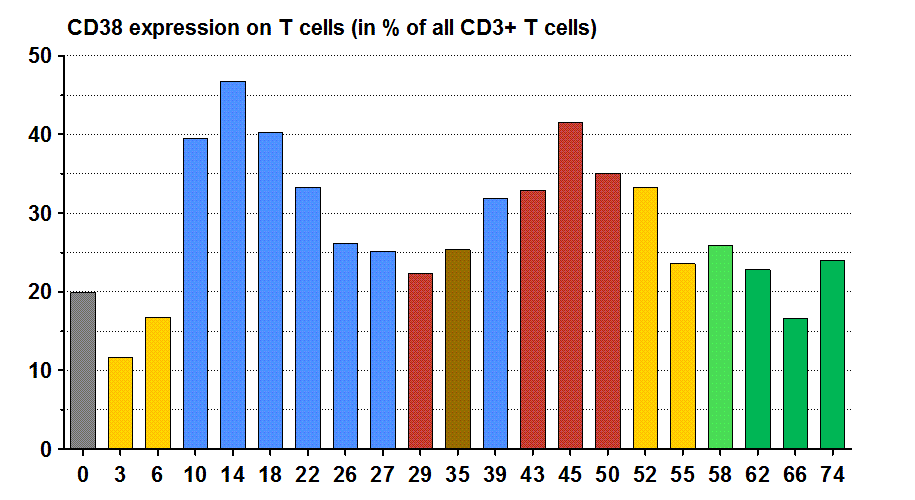
**

**Supplementary Figure 2: Multimodal therapy of glioblastoma has impact on the expression of the activation marker CD38 on T cells.** The graph shows the CD38 expression on T cells in percentage of all CD3+ T cells. The color code represents the last applied therapy or pathological finding (red) and was applied according to **Figure 1**.


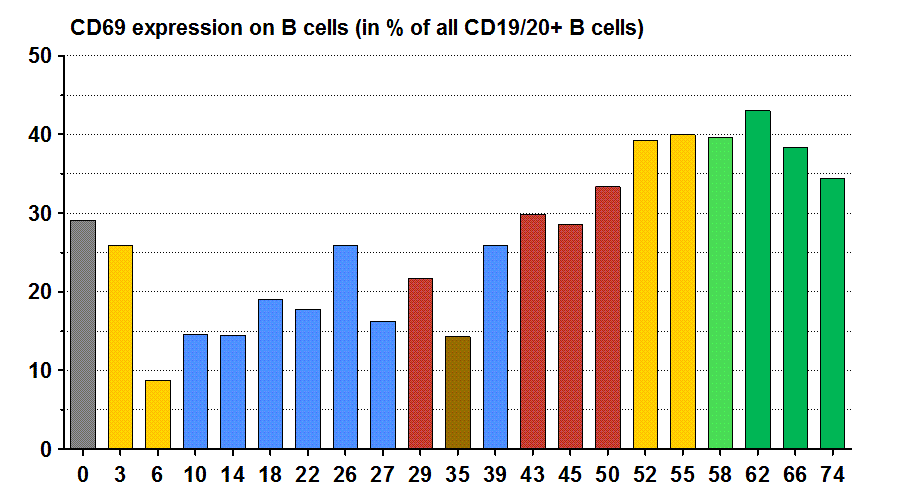


**Supplementary Figure 3: Multimodal therapy of glioblastoma has impact on the expression of the activation marker CD69 on B cells.** The graph shows the CD69 expression on B cells in percentage of all CD19/CD20+ B cells. The color code represents the last applied therapy or pathological finding (red) and was applied according to **Figure 1**.
